# Synthesis of Star 6-Arm Polyethylene Glycol-Heparin Copolymer to Construct Anticorrosive and Biocompatible Coating on Magnesium Alloy Surface

**DOI:** 10.3389/fbioe.2022.853487

**Published:** 2022-02-09

**Authors:** Qingxiang Hong, Hualan Zhou, Yuxin Cheng, Minhui Yang, Qiuyang Zhang, Sen Liu, Qingping Xiong, Changjiang Pan

**Affiliations:** ^1^ Faculty of Mechanical and Materials Engineering, Jiangsu Provincial Engineering Research Center for Biomaterials and Advanced Medical Devices, Huaiyin Institute of Technology, Huai’an, China; ^2^ The Affiliated Huai’an Hospital of Xuzhou Medical University, Huai’an, China; ^3^ Faculty of Chemical Engineering, Huaiyin Institute of Technology, Huai’an, China

**Keywords:** magnesium alloy, corrosion resistance, biocompatibility, polyethylene glycol, heparin

## Abstract

Magnesium alloy has become a research hotspot of the degradable vascular stent materials due to its biodegradability and excellent mechanical properties. However, its rapid degradation rate after implantation and the limited biocompatibility restrict its application in clinic. Constructing a multifunctional bioactive polymer coating on the magnesium alloys represents one of the popular and effective approaches to simultaneously improve the corrosion resistance and biocompatibility. In the present study, the copolymer of 6-arm polyethylene glycol and heparin (PEG-Hep) was successfully synthesized and then immobilized on the surface of chitosan (Chi)-modified magnesium alloy surface through electrostatic interaction to improve the corrosion resistance and biocompatibility. The results of attenuated total reflection Fourier transform infrared spectroscopy (ATR-FTIR), X-ray photoelectron spectroscopy (XPS) and scanning electron microscopy showed that a dense and compact coating was created on the magnesium alloy surface. The coating displayed excellent hydrophilicity. At the same time, the as-prepared coating can significantly not only improve the corrosion potential, reduce the corrosion current and the pH changes of the immersion solution, but also keep a relatively intact surface morphology after immersing in simulated body fluid solution for 14 days, demonstrating that the coating can significantly improve the corrosion resistance of the magnesium alloy. Moreover, the magnesium alloy with PEG-Hep coating exhibited excellent hemocompatibility according to the results of the hemolysis rate and platelet adhesion and activation. In addition, the modified magnesium alloy had a good ability to promote the endothelial cell adhesion and proliferation. Therefore, the PEG-Hep multifunctional coating can be applied in the surface modification of the biodegradable magnesium alloy stent to simultaneously improve the corrosion resistance and biocompatibility.

## Introduction

Vascular disease caused by the cardiovascular stenosis has become one of the highest incidences and mortality rates in the world (Lv et al., 2017; He et al., 2019). Although the stent insertion has significantly reduced the mortality rate (Sigwart et al., 1987; Qi et al., 2013), the inferior biocompatibility of the current non-biodegradable stent and the complications of the late thrombosis and the delayed endothelium healing caused by the released drug from the polymer coating on stent often lead to the implantation failure (Torii et al., 2020). In recent years, the biodegradable stent made from the biodegradable polymers and metals has attracted more and more attention thanks to their acceptable biodegradability and biocompatibility (Bowen et al., 2015; Zhou et al., 2019; Shi et al., 2020). Due to its limited toughness and strength, the polymer stent must have a larger stent strut thickness to produce the mechanical properties similar to metal stent. Moreover, the degradation products of the polymer stents often lead to inflammation, resulting in the occurrence of complications such as late thrombosis and delayed endothelial healing and finally leading to in-stent restenosis. Therefore, the biodegradable metal stents have received more and more attention. Due to it good mechanical and biodegradable properties, the magnesium alloy has become the research hotspot of the biodegradable cardiovascular stents (Zhang et al., 2021a; Yang et al., 2021). However, the rapid degradation *in vivo* and the limited biocompatibility are still great challenges for its clinical application.

Because the corrosion resistance and biocompatibility of the magnesium alloys are closely related to their surface properties, surface modification represents one of the basic methods to reduce the corrosion rate and enhance the bioactivities of the magnesium alloys. At present, three strategies have been employed to improve the corrosion resistance of the magnesium alloys. One is to produce a chemical conversion layer on the surface by chemical treatment or electrochemical treatment (Kröger et al., 2018; Jiang et al., 2019; Yan et al., 2019), however, the biological activities of the chemical conversion layers need to be improved. The second strategy is to improve the corrosion resistance of magnesium alloy by changing the surface microstructure (such as ion implantation, surface heat treatment, etc.), but its biocompatibility is still very limited. The third is to construct a covering layer on the surface, such as surface self-assembly (Pan et al., 2016) and layer by layer self-assembly (Zhang et al., 2021b), deposition of inorganic coating (Li et al., 2020a), formation of layered double hydroxide (Guo et al., 2018), preparation of polymer coating, etc. The covering layer not only effectively improves the corrosion resistance, but also enhances the biocompatibility to a certain extent. However, due to the lack of sufficient biological activities on the surface, it may still lead to coagulation and delayed healing of the endothelium.

Among the strategies to improve the corrosion resistance and biocompatibility of the magnesium alloys, the polymer coating does not change the matrix properties, and it not only has good degradation performance and biocompatibility, but also can regulate cell behaviors to some degree and be used for drug loading. It has become one of the most effective methods to improve the corrosion resistance of the magnesium alloys. Generally speaking, the polymer coatings mainly include sol-gel polymers, synthetic polymers and natural polymers. The sol-gel polymer coating is mainly produced by the hydrolysis of organosilicon compounds to form a firmly bonded polymer coating with substrate, which can significantly improve the corrosion resistance and blood compatibility to some degree, as well as promote growth of vascular endothelial cells (Liu and Xi, 2016). Synthetic polymers mainly include polylactic acid (PLA), polyglycol-lactide (PLGA), polycaprolactone (PCL) (Jiang et al., 2017), polyurethane (PU) (Wang et al., 2019), polycarbonate (PC) (Pan et al., 2022) and plasma polymerization coatings (Qi et al., 2016), etc. Although the synthetic polymer coatings can significantly improve the corrosion resistance of the magnesium alloys, the biocompatibility is relatively limited. Therefore, synthetic polymer coatings often need to be further modified by the surface biofunctionalization or drug loading to improve the biocompatibility. Natural polymers mainly include chitosan (Jiang et al., 2016), hyaluronic acid (Li et al., 2020b), silk fibroin (Xu et al., 2019), etc. Natural polymers have better biocompatibility due to their biomimetic properties, but they still cannot fundamentally improve the biocompatibility of the magnesium alloys. Therefore, the biocompatibility of natural polymer coatings still needs to be improved by other surface modification methods.

Heparin is a polysaccharide substance with excellent blood compatibility and it has been widely used for the surface modification of the blood-contacting biomaterials. Heparin can not only improve the blood compatibility, but also promote the growth of endothelial cells to some extent (Pan et al., 2014), even selectively promote endothelial cell growth (Liu et al., 2017; Zhang et al., 2018). 6-arm polyethylene glycol (PEG) is a kind of polymer with excellent hydrophilicity and anti-biofouling ability, which can also provide a good cell growth microenvironment and inhibit nonspecific protein adhesion. Therefore, if 6-arm polyethylene glycol and heparin copolymer (PEG-Hep) is introduced on the magnesium alloy surface, it should not only improve the corrosion resistance, but also enhance the blood compatibility and promote endothelial cell growth. To this end, the copolymer of 6-arm polyethylene glycol and heparin (PEG-Hep) was firstly synthesized. Then, the magnesium alloy was treated by hydrofluoric acid followed by the deposition of a polydopamine layer on the surface. The chitosan was immobilized on the surface via Michael addition reaction between polydopamine coating and amine groups of chitosan to produce the positive-charged surface. Finally, the synthesized copolymer (PEG-Hep) was immobilized on the surface by electrostatic adsorption. The results *in vitro* demonstrated that the coating can significantly improve the corrosion resistance and biocompatibility of the magnesium alloy.

## Materials and Methods

### Synthesis of Polyethylene Glycol-Hep

**SCHEME 1:**
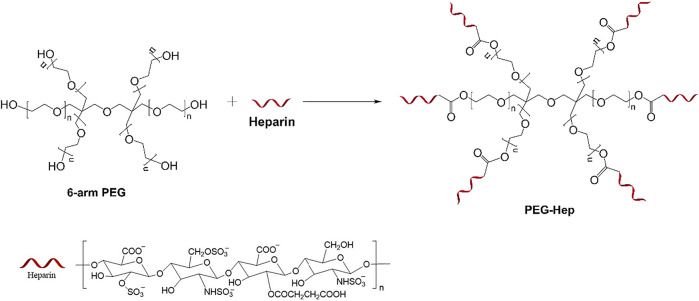
The synthesis route of PEG-Hep.

The synthesis route of PEG-Hep was shown in Scheme 1. In brief, 50 ml tetrahydrofuran was firstly added into a 100 ml single mouth flask and then 10 g 6-arm polyethylene glycol (PEG, Sigma-Aldrich Shanghai, China) and 0.5 g heparin (Sigma-Aldrich Shanghai, China) were added. After thoroughly stirring in ice bath for 25 min, 0.03 g DMAP (4-dimethylamino-pyridine) and 0.2 g EDC (N-(3-Dimethylaminopropyl)-N′-ethylcarbodiimide hydrochloride, Sigma-Aldrich Shanghai, China) was added to react 12 h at room temperature under stirring. After precipitating by the ice ethanol and filtration, the products were dried in the vacuum oven. The molecular structure of PEG-Hep was determined by nuclear magnetic resonance hydrogen spectrum (^1^H-NMR) and ATR-FTIR.

### Surface Modification

AZ31B magnesium alloy with the diameter of 12 mm was cut into 3 mm thickness slices, followed by successively polishing using 400#, 800#, 1000#, 1500#, 2000# sandpapers. The polished samples (Mg) were ultrasonically cleaned in acetone, ethanol, deionized water for 5 min, respectively. The sample was immersed in a 12 mol/L hydrofluoric acid (HF) solution for 15 min. After washed and dried, the HF treated sample (Mg-HF) was immersed in 2 mg/ml dopamine solution (Tris buffer, pH8.5) for 12 h at room temperature. The chitosan was then immobilized on the polydopamine-modified sample (Mg-DA) by putting the sample into the chitosan solution (0.2% acetic acid, pH 5) for reacting 2 h. The chitosan-modified sample (Mg-Chi) was immersed into 2 mg/ml PEG-Hep solution to adsorb heparin through electrostatic interaction, and the resulting sample was denoted as Mg-PEG-Hep.

### Surface Characterization

The surface chemical structures of the samples were characterized by ATR-FTIR (TENSOR27, Bruker of Germany) with a scanning range of 4,000–650 cm^−1^ at room temperature, and the elemental analysis were performed by X-ray photoelectron spectroscopy (XPS, Quantum 2000; PHI Co., Chanhassen, MN) with Al Kα source (1486.6 eV). The morphology and surface wettability of the samples were observed by scanning electron microscopy (SEM, FEI, Quata 250, United States) and contact angle meter (KR ü SS GmbH, Germany) at room temperature, respectively. In order to ensure the accuracy of the water contact angle, three parallel samples were measured to calculate the average value.

### Corrosion and Degradation Behaviors

The potentiodynamic polarization curves of the different samples were measured by a CHI660D electrochemical workstation (CHI Instruments, Inc., Shanghai, China) using a standard three-electrode system. The corrosive medium is simulated body fluid (SBF, composition: NaCl 8 g/L; KCl 0.4 g/L; NaHCO_3_ 0.35 g/L; CaCl_2_ 0.14 g/L; Na_2_HPO_4_ 0.06 g/L; KH_2_PO_4_ 0.06 g/L; MgSO_4_·7H_2_O 0.01 g/L; glucose 1 g/L). The sample, saturated Ag/AgCl electrode and platinum electrode are working electrode, reference electrode and auxiliary electrode, respectively. Before the test, the sample was sealed with the silicone rubber using a copper wire as the conductor. The exposed area was 1 cm^2^. The sample was immersed into 100 ml SBF solution for 10 min to achieve a stable open circuit potential before the polarization test. The potentiodynamic polarization test was carried out at a scanning rate of 1 mV/s. The corrosion current and corrosion potential were determined according to the polarization curve.

In order to further determine the corrosion behaviors, the pristine magnesium alloy and the modified samples were immersed in SBF solution at 37°C for 1 day, 3, 7 and 14 days, respectively. Before the experiment, the sample needs to be encapsulated with silicone rubber. The sample was placed in a tube containing 20 ml SBF, and the medium was changed every 2 days. At the predetermined times, the sample was taken out, cleaned and dried, and the surface morphology was observed by a scanning electron microscopy.

At the same time, the degradable behaviors of the different samples were characterized by immersing experiment. Before testing, the samples were all encapsulated and then put them into 20 ml SBF solution with the initial pH of 7.4. The pH values of the solutions were measured by a pH meter at least three times for each time. Then the average value was obtained.

### Blood Compatibility

#### Hemolysis Assay

Fresh whole blood containing sodium citrate as an anticoagulant was legally obtained from a healthy volunteer. The blood was centrifuged at 1500 rev/min for 10 min to obtain the red blood cells. The concentration of the red blood cells was adjusted to 2% by physiological saline, and 2 ml solution was added to each sample to incubate 3 h at 37°C. Distilled water and normal saline were used to prepare 2% erythrocyte suspension as positive control and negative control, respectively. 1 ml red blood cell solution was taken from each sample and centrifuged 5 min at 3,000 r/min. 200 μL of the supernatant was taken and placed into a 96-well plate, the absorbance was measured at 450 nm by a microplate reader (Bio-Tek, Eons). The following formula was used to calculate the hemolysis rate.

R=(A−C1)/(C1−C2)×100

where, A, C1, C2 are the absorbance of the sample, negative and positive control, respectively.

#### Platelet Adhesion

The anticoagulated fresh whole blood was legally obtained from a healthy volunteer and then centrifuged at 1500 rpm for 10 min to collect the platelet rich plasma (PRP). 200 μL platelet-rich plasma was dropped on each sample to incubate 2.5 h at 37°C. The samples were washed twice by saline, followed by fixing 3 h at 4°C using 2.5% glutaraldehyde solution. Finally, the samples were dehydrated with 50, 70, 90, 100% ethanol solutions gradually, each step for 15 min. After dried in air, the attached platelets were observed by scanning electron microscopy.

### Endothelial Cell Behaviors

#### Endothelial Cell Adhesion

Before the experiment, the sample was sealed with silicone rubber and sterilized under an ultraviolet light. The samples were placed into 24-well plate and then 0.5 ml of 5×10^4^ cell/mL endothelial cells (EVC304, Cobioer, Nanjing, China) and 1.5 ml of cell culture medium were added into each sample. After incubating 6 and 24 h at 37°C and 5% CO_2_, respectively, the samples were rinsed twice by physiological saline. The attached cells were fixed with 2.5% glutaraldehyde at 4°C for 3 h. After rinsing twice with physiological saline, the cells were successively stained by rhodamine (PBS, 10 μg/ml) for 20 min and 4, 6-diamidino-2-phenylindole (DAPI, UP water, 500 ng/ml) for 10 min, respectively. After rinsed and dried, the fluorescent images of the attached endothelial cells were acquired by an inverted fluorescent microscopy (Carl Zeiss A2 inverted).

#### CCK-8 Assay

Before the experiment, the sealed samples were placed into 24-well plate to sterilize 24 h under an ultraviolet light. Then 0.5 ml of 5×10^4^ cell/mL endothelial cells and 1.5 ml of cell culture medium were added into each sample and incubate at 37°C and 5% CO_2_ for 6 and 24 h, respectively. After that, the samples were transferred into another new plate, and 0.5 ml 10% CCK-8 solution was added into each well for incubating 3.5 h. Finally, 200 μL CCK-8 solution was taken from each well and placed in a 96-well plate, and the absorbance at 450 nm was measured by a microplate reader (Bio-Tek Eons).

### Statistical Analysis

All the data of the water contact angle, pH changes, hemolysis rate and CCK-8 values in this paper were expressed by mean ± SD, and the statistical analyses were performed using SPSS 12.0. Statistically significant differences were determined by one way analysis of variance (ANOVA). A probability value (*p*) of less than 0.05 was considered to be statistically significant.

## Results and Discussion

### Structural Analysis of Polyethylene Glycol-Hep

**FIGURE 1:**
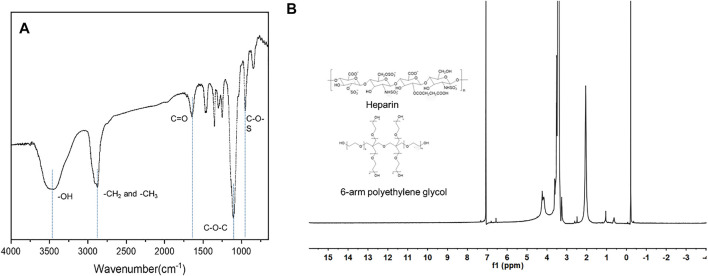
The ATR-FTIR **(A)** and ^1^H-NMR **(B)** of PEG-Hep.

The chemical structure of the synthesized PEG-Hep was characterized by ATR-FTIR and hydrogen nuclear magnetic resonance spectroscopy (^1^H-NMR). The results are shown in Figures 1A,B, respectively. For ATR-FTIR (Figure 1A), a broad absorption band appeared at 3,500 cm^−1^, which is mainly due to the stretching vibration of -OH. The peaks of CH_2_ and CH_3_ at 2,980 cm^−1^ and 2,890 cm^−1^ can be clearly observed, and the peaks of C-O-C at 1275–1020 cm^−1^ can also be detected. They are all characteristic peaks of PEG. The peaks of C-O-S at 842 cm^−1^ and C=O at 1700 cm^−1^ suggested that heparin was successfully introduced. In ^1^H-NMR spectra, the integral intensity ratio from low field to high field is 2:3, combined with the results of ATR-FTIR, it can be known that the substance has the chemical structure of CH_2_-CH_3_, and the OH peak appeared at 3.42 ppm. The H1 of N-sulfated glucosamine and the methyl of N-acetylglucosamine in heparin appear at 3.27 ppm and 2.05 ppm, respectively. All these results indicated that the PEG-Hep copolymer was successfully obtained.

### Surface Characterization of the Modified Magnesium Alloy

**FIGURE 2:**
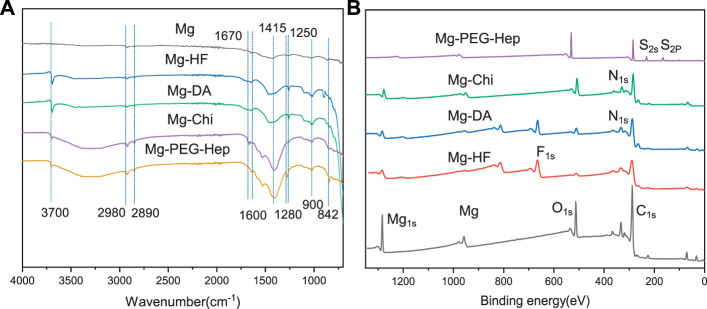
The ATR-FTIR **(A)** and XPS **(B)** spectra of the different samples.

The surface chemical structures of the samples were firstly characterized by ATR-FTIR, and the results are shown in Figure 2A. There was no infrared absorption on the pristine magnesium alloy surface, suggesting that there were no chemical groups on the blank magnesium alloy surface. After hydrogen fluoride treatment, a chemical layer of MgF_2_ was produced on the surface, leading to the occurrence of the sharp peak at 900 cm^−1^ (Pan et al., 2017). The Mg-DA sample had new absorption peaks at 1250 cm^−1^ and 1600 cm^−1^, which are caused by the stretching vibration of the C-O bond and the bending vibration and shearing vibration of the N-H. After chitosan was grafted on the surface, two new absorption peaks appeared at 1670 cm^−1^ and 1415 cm^−1^, namely C=O and C-N, indicating that chitosan was successfully immobilized on the surface of Mg-DA sample. The Mg-PEG-Hep sample showed a sharp peak corresponding to the hydroxyl group at 3,700 cm^−1^, and the peaks at 2,980 cm^−1^ and 2,890 cm^−1^ were the appearance of CH_2_ and CH_3_, respectively. The characteristic peak at 842 cm^−1^ and 1280 cm^−1^ were the appearance of C-O-S and S=O, respectively, indicating that PEG-Hep was successfully introduced on the Mg-Chi surface.

XPS was utilized to further detect the surface atomic concentrations of the different samples, and the results are shown in Figure 2B and Table 1. In addition to Mg_1s_, the blank magnesium alloy surface had C_1s_ and O_1s_ peaks, indicating that the magnesium surface was oxidized and C contamination appeared. After the hydrogen fluoride treatment, the oxygen concentration was reduced, and F_1s_ peak appeared, suggesting that the MgF_2_ chemical conversion layer on the surface was produced and replaced the previously oxide layer. The decreased O_1s_ peak and the occurrence of the N_1s_ peak on Mg-DA surface indicated that the polydopamine layer was formed on the surface, which can provide the reaction sites for the subsequent reaction. After grafting chitosan, the C_1s_, O_1s_ and N_1s_ peaks were all significantly enhanced. For Mg-PEG-Hep, the C_1s_ and O_1s_ peaks on the surface were significantly enhanced, the N_1s_ content was reduced, and S_2p_ and S_2s_ appeared, indicating that PEG-Hep was successfully introduced on the surface. In conclusion, it can be inferred that DA, Chi and PEG-Hep were successfully introduced on the magnesium alloy.

**TABLE 1:** The surface atom percentages (wt%) of the different samples characterized by X-ray photoelectron spectroscopy (XPS).

Samples	Mg	O	C	S	N	F
Mg	47.3	46.20	6.5	-	-	-
Mg-HF	10.55	23.97	9.3	-	-	56.18
Mg-DA	7.31	35	40.25	-	5.44	12
Mg-Chi	2.97	41.53	48.38	-	7.12	-
Mg-PEG-Hep	0.18	44.04	50.31	5.19	0.28	-

**FIGURE 3:**
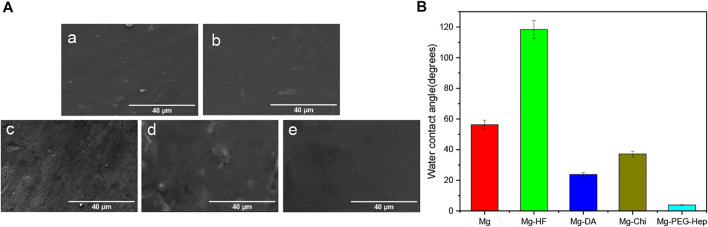
**(A)** SEM images of **(a)** Mg, **(b)** Mg-HF, **(c)** Mg-DA, **(d)** Mg-Chi, **(e)** Mg-PEG-Hep, **(B)** The water contact angles of the different samples.

The surface morphologies of the samples were characterized by scanning electron microscopy (SEM). As shown in Figure 3A, the surface of the magnesium alloy is relatively flat after polishing. After the hydrogen fluoride treatment, the MgF_2_ layer was formed on the magnesium alloy according to ATR-FTIR and XPS, which can play protective role to prevent the corrosion of the magnesium alloys (Da Conceicao et al., 2010), and initially improve the corrosion resistance of the magnesium matrix. Dopamine (DA) is a kind of high adhesion protein inspired by mussel foot. The dopamine molecule can self-polymerize to polydopamine (PDA) in air which has a strong binding force with a variety of materials (Waite, 1983; Liu et al., 2014a). Moreover, it can also react with substances containing amino and thiol groups. From the XPS results, it can be known that the polydopamine was successfully immobilized on the Mg-HF sample. Compared with the Mg-HF, the surface of Mg-DA was rougher, which may be caused by the time-consuming immersion process and a large amount of PDA agglomeration (Ball et al., 2012; Wang et al., 2014). After grafting chitosan, white particles appeared on the surface, which is a common feature of chitosan films (Zhai et al., 2018). The positive charges of chitosan can absorb the negative-charged heparin, so that PEG-Hep can be successfully immobilized on the surface of Mg-Chi. The surface of Mg-PEG-Hep was smoother than Mg-Chi, which can protect the magnesium matrix and thus improve the corrosion resistance.

Surface hydrophilicity plays an important role in the biocompatibility of the biomaterials. Good hydrophilicity can prevent the nonspecific protein adhesion and thus improve the blood compatibility and provide good cell compatibility. The hydrophilicity of the magnesium alloy before and after surface modification was evaluated by water contact angle, and the results are shown in Figure 3B. The water contact angle of the original magnesium was 56.2°, exhibiting limited hydrophilicity. The original magnesium surface had no hydrophilic groups (as shown in Figures 2A,B) and the surface was smooth (as shown in Figure 3A), resulting in the limited hydrophilicity. After the hydrogen fluoride treatment, the water contact angle increased to 118.4°. Studies have shown that the MgF_2_ chemical layer formed after the hydrofluoric acid treatment has the characteristics of low water solubility (Makkar et al., 2018), which is the reason for the increase in the water contact angle. After polydopamine deposition, the water contact angle was reduced to 23.8° because of the introduction of hydrophilic amine groups, and the enhanced surface roughness (as shown in Figure 3A) also contributed to the better hydrophilicity. Chitosan is a hydrophilic polymer (Ludwiczak and Mucha, 2010), but the Michael addition reaction between the polydopamine coating and the amino groups in chitosan formed a dense layer, resulting in a slight decrease in hydrophilicity, and thus the water contact angle was increased to 37.1°. Heparin is a natural polysaccharide, which has rich hydrophilic groups such as hydroxyl, carboxyl and sulfonic acid groups, therefore, the water contact angle of Mg-PEG-Hep was reduced to 3.9°, displaying the superhydrophilicity.

### Corrosion Behaviors

**FIGURE 4:**
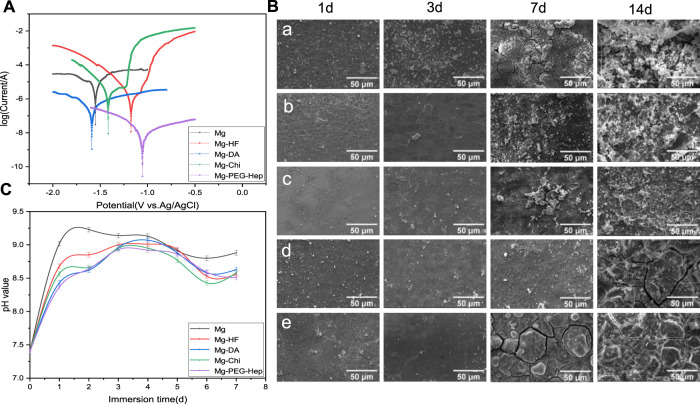
**(A)** Potentiodynamic polarization curves of the different samples. **(B)** Typical SEM images of the samples immersed in SBF for 1, 3, 7, and 14 d **(a)** Mg, **(b)** Mg-HF, **(c)** Mg-DA, **(d)** Mg-Chi, **(e)** Mg-PEG-Hep, **(C)** pH values of the different samples immersed in SBF for 7 days.

The potentiodynamic polarization curves of the magnesium alloy before and after modification in the simulated body fluid are shown in Figure 4A, and the corresponding corrosion potential and current are summarized in Table 2. Generally speaking, a high corrosion potential means that the material is thermodynamically more stable and resistant to corrosion, while a low corrosion potential means that the corrosion tendency is lager and the corrosion resistance could become worse. The surface of the unmodified magnesium alloy is active, therefore, the corrosion potential (-1.57 V) was the lowest and the corrosion current (1.419 × 10-4A·cm^−2^) was the highest among all samples, indicating that the corrosion resistance of unmodified magnesium alloy was the worst. After hydrogen fluoride treatment, a MgF_2_ layer was formed. Lee et al. proved that the MgF_2_ layer on the magnesium alloy has the advantages of good compactness and high bonding strength with the substrate (Makkar et al., 2018). The dense film layer is not conducive to the penetration of electrolytes, and the poor hydrophilicity can prevent water adhesion, consequently, it can reduce the corrosion rate of the magnesium alloy. After the polydopamine deposition, the polymer layer on the magnesium alloy can isolate the substrate from the corrosive medium and thus effectively protect the magnesium matrix, and the corrosion current was further reduced to ×3.37010^−7^ A cm^−2^. At the same time, the self-polymerized dopamine-coated catechol can also undergo a Michael addition reaction with the amine group of chitosan, thus chitosan was grafted on the magnesium alloy surface. After grafting chitosan, the positive charges of chitosan can adsorb anions and hinder the direct erosion of anions to the magnesium matrix, leading to the improved corrosion resistance. At the same time, the increase of the polymer layer thickness can also improve the corrosion resistance. After PEG-Hep immobilization, the anions of heparin can combine with the positive charges of chitosan, making the surface exhibit electronegativity to prevent the adsorption of anions, so that the corrosion resistance of the magnesium alloy was further improved, and the corrosion potential increased to −1.055 V, the corrosion current was reduced to 6.650 × 10^−9^ A cm^−2^. It can be concluded that the PEG-Hep coating can significantly improve the corrosion resistance of the magnesium alloy. It can be seen from Figure 3A that the surface film layer was relatively dense, which made the magnesium alloy difficult to corrode in the SBF and may achieve the purpose of long-term protection of the magnesium alloy.

**TABLE 2:** Corrosion potential and corrosion current densities of the different samples.

Samples	Ecorr/V	Icorr/A·cm^−2^
Mg	−1.570	1.419 × 10^−4^
Mg-HF	−1.175	1.177 × 10^−6^
Mg-DA	−1.589	3.370 × 10^−7^
Mg-Chi	−1.417	3.071 × 10^−6^
Mg-PEG-Hep	−1.055	6.650 × 10^−9^

The corrosion behaviors of the different samples were further evaluated by immersion test. The samples were immersed in SBF solution for 1 day, 3, 7 and 14 days, respectively, and then the surface morphologies of the samples before and after immersion were observed by scanning electron microscopy. The typical surface morphologies of the different samples after immersing different time were shown in Figure 4B. Elemental analysis was also performed by EDX after immersion and the results are listed in Table 3. It can be seen that the small cracks appeared on the surface of the magnesium alloy after soaking 1 day, because the pristine magnesium alloy had loose magnesium oxide, which cannot resist the erosion of anionic ions in SBF, leading to the poor corrosion resistance. Hydrogen gas was generated during pretreatment with hydrogen fluoride, resulting in a few microcracks on the Mg-HF surface. The released hydrogen during the soaking process and the presence of the corrosive Cl^−^ can weaken the adhesion between PDA and Mg, resulting in the appearance of small corrosion pits on the Mg-DA surface. However, no cracks can be observed on the Mg-Chi and Mg-PEG-Hep surfaces, indicating that the introduction of chitosan and PEG-Hep can significantly enhance the corrosion resistance of the magnesium alloy. After 3 days of immersion, the surface cracks of the magnesium alloy increased significantly. Studies have shown that the corrosion rate of the magnesium alloys is faster at 3 days as compared to 14 days (Makkar et al., 2018). On one hand, the initial larger area exposed to the solution could promote the exothermic reaction and increase the corrosion rate; on the other hand, the higher concentration of Cl^−^ and other salts may also cause the corrosion rate to increase. The surface of other modified samples was basically unchanged at 1 day, and no cracks occurred, indicating that dopamine, chitosan and PEG-Hep coatings were beneficial to improve the corrosion resistance of magnesium alloy to the different degree. On the 7th day, the corrosion on the magnesium surface became serious, and the cracks and corrosion products increased significantly. The corrosion of other samples was also more serious than before. However, there were only microcracks on the Mg-Chi and MG-PEG-Hep surface because they could cover the magnesium alloy surface and protect the magnesium alloy matrix from corrosion. After being immersed 14 days, it can be seen that white granular substances appeared on all sample surfaces (Figure 4B), and it was found that the main component may be hydroxyapatite (HA, Ca_10_ (PO_4_) _6_ (OH)_2_) (Ly and Yang, 2019). As shown in Table 3, P and Ca elements appeared on the surfaces of all samples, which may be Ca-P particles formed on the surface. The surface corrosion of Mg was more serious, and the content of Mg decreased, and the content of O and C increased, indicating that these corrosion products may be carbonate. The increase of P element content on the Mg-HF surface indicates that the MgF_2_ coating on the surface may be damaged. After the fixation of dopamine, the content of P and Mg further increased, and the small pits on the surface of the PDA coating caused serious corrosion of the samples. The changes of elements in other samples were not different from Mg. During the immersion of Mg-Chi, the chitosan film was slightly corroded, and a small amount of corrosion products appeared, so the elements C and Mg basically did not change, and the content of O increased. Mg-PEG-Hep surface coating had the best integrity, less corrosion products and the highest Mg content can be observed, indicating that it had the best corrosion resistance. On one hand, the coating can act as an isolating barrier between magnesium alloy and SBF, and on the other hand, heparin with negative charge properties can inhibit anion erosion.

**TABLE 3:** Surface element contents of the different samples after immersing in SBF for 14 days.

Samples	Mg	O	C	Ca	P		
Mg		7.13	40.37	22.65	26.45	3.58
Mg-HF	5.03	42.78	42.62	5.35	4.21	
Mg-DA	6.12	36.69	36.47	13.65	7.07	
Mg-Chi	5.22	53.83	24.51	9.43	7.01	
Mg-PEG-Hep	9.50	20.16	65.69	0.60	4.05	

The pH in the human body is about 7.4, which is weakly alkaline. Too high pH will damage the normal growth microenvironment of cells. The high corrosion rate of magnesium alloy can produce a large amount of OH^−^, resulting in the increase of pH. Therefore, the pH changes of the different samples soaked in SBF for 7 days were measured and the results are shown in Figure 4C. The pH value of all samples increased rapidly on the first day, among which Mg increased the fastest (from the 7.4–9.02), indicating that its corrosion resistance was the worst. After 2 days, the pH value changed slowly and became steady, implying that the degradation rate became slower. During the immersion process, the pH value of Mg was always the highest, so its corrosion resistance was the worst. After hydrogen fluoride treatment, a chemical conversion layer of MgF_2_ was formed. Yuan et al. proved that the NaHCO_3_, NaCl and other components in SBF can effectively prevent the dissolution of MgF_2_ (Mao et al., 2013), so the pH value change tends to be flat. After grafting chitosan, the positive charge of the amine group can prevent the corrosion of Cl^−^ and other anions, so it can have a long-term protective effect on the magnesium matrix. After 4 days of immersion, a dynamic equilibrium was reached between the dissolution and corrosion products of the samples, and the corrosion rate decreased, so the pH value decreased, which was consistent with the results of the immersion experiment. The pH values of the Mg, Mg-HF, Mg-DA, and Mg-Chi samples increased slightly after being soaked for 6 days. This may be because the corrosion products had no protective effect on the magnesium matrix over time. After immersing 7 days, the pH value of Mg-PEG-Hep was the lowest. The positive charges of chitosan can react with the negative charge of heparin to form a dense protective layer, as shown in Figure 4B, which can effectively inhibit the corrosion of the magnesium alloy.

### Blood Compatibility

**FIGURE 5:**
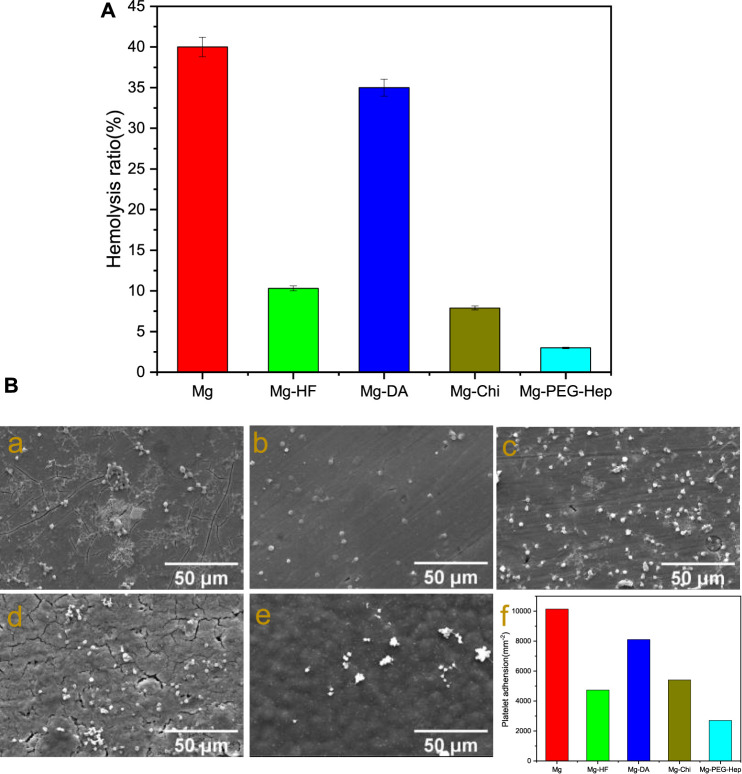
**(A)** Hemolysis rate of the different samples. **(B)** SEM images of platelet adhesion on the different samples. **(a)** Mg, **(b)** Mg-HF, **(c)** Mg-DA, **(d)** Mg-Chi, **(e)** Mg-PEG-Hep, **(f)** The number of the attached platelets on the different samples.

Human blood is mainly composed of red blood cells, white cells, platelets and so on. When the stent enters the human body, the red blood cells may be damaged and even ruptured, resulting in severe hemolysis. National standards stipulate that the hemolysis rate of biomaterials or devices should be less than 5%. The hemolysis rates of the different samples are shown in Figure 5A. Due to the limited corrosion resistance, the blank magnesium alloy can produce a large amount of OH^−^ during the corrosion process, which will increase the local pH. High pH will increase the binding capacity of hemoglobin and membranes, leading to the increase of hemolysis rate. Severe hemolysis can cause the hemolysis rate of magnesium alloys to reach as high as 40%. After hydrogen fluoride treatment, the hemolysis rate was 10.3%. Studies have shown that the hemolysis rate of MgF_2_ formed after hydrofluoric acid treatment is 10.1% (Mao et al., 2013). This is because fluoride itself is biocompatible, and the increased corrosion resistance can also reduce the release of OH^−^. After the deposition of polydopamine, the hemolysis rate increased rapidly to 35%. The research showed that although the polydopamine layer could slow down the corrosion of the material (as shown in Figure 4A), however, due to the amine groups on its surface, more platelets and red blood cells adhere to the surface, resulting in the poor blood compatibility (Chen et al., 2018). After grafting chitosan, the hemolysis rate was about 7.9%, the negatively charged glycoprotein on the surface of erythrocytes can combine with the positively charged amino group of chitosan, resulting in the decrease of hemolysis rate, but it is still larger than 5%. After PEG-Hep was introduced, the hemolysis rate decreased to 3.8%. It can be considered that heparin can not only enhance the activity of thrombin III, thereby indirectly exerting anticoagulant effect, but also can improve the activities of protein C and stimulate endothelial cells releasing anticoagulant and fibrinolytic substances (Wu, 1996), so the hemolysis rate of Mg-PEG-Hep can meet the requirement of the national standard for hemolysis rate.

Another way of evaluating blood compatibility is platelet adhesion. When the stent enters the human body, platelet aggregation and activation may occur to promote the production of thrombin, thereby forming a thrombus. Therefore, platelets are considered to be one of the important causes of thrombus formation. The SEM images of platelet adhesion and the amount of platelets on the different samples are shown in Figure 5B. The platelets on the magnesium alloy were mostly in an aggregated state, and most of them had pseudopodia exhibiting a spreading state, indicating that the blood compatibility of the magnesium alloy was poor. After hydrogen fluoride treatment, a chemical layer of MgF_2_ was formed. Mao et al. proved that MgF_2_ has good anti-platelet adhesion ability (Mao et al., 2013), and the improvement of corrosion resistance (as shown in Figure 4A) can also reduce the platelet adhesion. Wei et al. (2011) and Ma et al. (2013) have also proved that the imine and quinine groups in the polydopamine layer can quickly adsorb proteins, leading to platelet adhesion and aggregation, therefore, compared with Mg-HF, the number of platelets adhered to Mg-DA was increased. In general, surfaces with good hydrophilicity have lower surface free energy which can prevent the adhesion of plasma proteins and thus inhibit platelet adhesion (Gao et al., 2019). After chitosan graft, the decreased hydrophilicity (as shown in Figure 3B) resulted in platelet aggregation, so the number of platelet adhesion did not decrease significantly. Heparin has excellent anticoagulant effect and can reduce platelet adhesion and thus only several round platelets can be found on Mg-PEG-Hep. Meanwhile, Mg-PEG-Hep had good surface wettability, which can preventthe non-specific protein adsorption, and thus further reduced the platelet adhesion and aggregation, so the number of the attached platelets decreased significantly, which also confirmed that Mg-PEG-Hep had good blood compatibility.

### Endothelial Cell Behavior

#### Cell Adhesion

**FIGURE 6:**
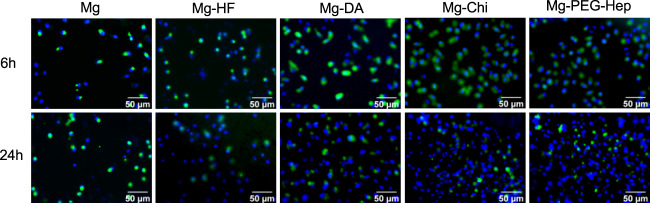
SEM images of the endothelial cells adhered on the different samples surfaces for 6 and 24 h, respectively.

The inner wall of normal human blood vessel is an endothelial layer composed of vascular endothelial cells. Vascular endothelium plays a very important role in maintaining the dynamic balance of human blood and preventing excessive proliferation of smooth muscle cells. When cardiovascular materials are implanted into human blood vessels, the surrounding endothelial tissue is damaged, which causes endothelial dysfunction. Therefore, for intravascular implant, the rapid formation of a vascular endothelial layer on the surface after the implantation is one of the important methods to solve the clinical complications of the cardiovascular implants. We first used endothelial cell adhesion to characterize the ability to promote endothelial growth. The fluorescent images of the endothelial cells cultured on the different samples for 6 and 24 h, respectively, are shown in Figure 6. Cells are very sensitive to environmental fluctuations, any small physical and chemical changes may lead to bad results (Witte et al., 2008; Xin et al., 2009). Magnesium can produce a large amount of OH^−^ during the corrosion process to cause local alkalization, and at the same time a large amount of the released Mg^2+^ may damage the cell growth environment. Consequently, with the extension of the culture time, the number of cells did not change significantly for the pristine magnesium alloy. As compared with Mg, Mg-HF had a slight increase in the number of cell adhesion after 6 and 24 h culture. It can be attributed to the improved corrosion resistance and the hydrophobic surface of the MgF_2_ layer. Studies have shown that endothelial cells can better adhere to hydrophobic surfaces (Dekker et al., 1993). Studies have shown that the polydopamine layer can effectively enhance the adhesion, proliferation and migration of endothelial cells (Yang et al., 2012; Liu et al., 2014b), and the hydrophilicity was also improved, so the number of endothelial cell adhesion was increased on Mg-DA. Chitosan is a natural polysaccharide polymer that can promote cell adhesion and proliferation. Okamoto et al. (2002) demonstrated that chitosan and its derivatives can stimulate endothelial cells to secrete the inflammatory cytokine Interleukin-8 (IL-8). IL-8 has vascular proliferation and chemotactic attraction to endothelial cells, thus can promote endothelial cell migration and proliferation (Koch et al., 1992; Ueno et al., 1999). Therefore, the number of endothelial cells on Mg-Chi was further increased. Heparin is a natural polysaccharide molecule, which can not only improve the blood compatibility of the material, but also promote the growth of endothelial cells (Gao et al., 2019). PEG and its derivatives have excellent inhibition of non-specific adhesion of proteins, platelets and cells, and contain a large number of hydrophilic groups (Banerjee et al., 2011; Alibeik et al., 2012; Kovach et al., 2014). In addition, the PEG-Hep coating can endow magnesium alloys with good corrosion resistance (as shown in Figure 4), which can significantly reduce the hydrogen bubbles and local alkalization caused by excessive corrosion of magnesium alloys, thereby improving endothelial cells growth. Therefore, Mg-PEG-Hep shows good biocompatibility, and the number of endothelial cell adhesion was the largest.

#### Cell Proliferation

**FIGURE 7:**
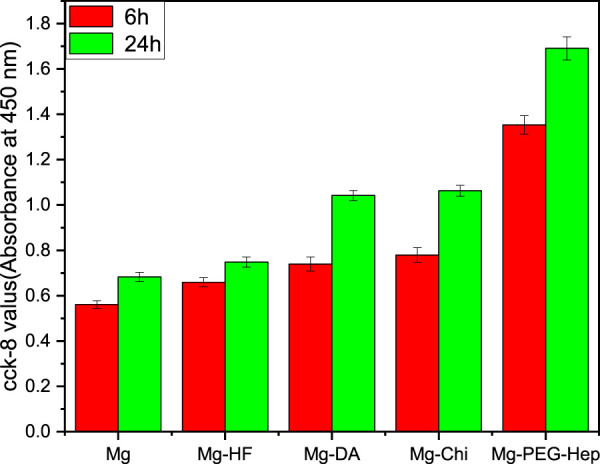
The CCK-8 results of the endothelial cells adhered on the different samples for 6 and 24 h.

The cell proliferation was characterized by CCK-8 assay. Generally speaking, the higher the CCK-8 value, the more the number of cells. The CCK-8 values of the different samples are shown in Figure 7. After 6 h incubation, the CCK-8 value of the magnesium alloy was the lowest. This may be due to the poor corrosion resistance of the blank magnesium, and the increase of pH value caused local alkalization, and at the same time it was accompanied by the generation of hydrogen bubbles, which was not conducive to cell growth. After hydrogen fluoride treatment, the hydrophobic Mg-HF surface lacks reactive groups and cannot bind cells through non-receptors, which is not conducive to cell adhesion. However, the hydrophobic surface may adsorb proteins in the culture medium, thereby promoting cell adhesion and proliferation (Pan et al., 2017), resulting in the increased CCK-8 values. After the deposition of the polydopamine, both the corrosion resistance and hydrophilicity increased, and a variety of active groups were also introduced, so the CCK-8 value increased significantly. Chitosan is a natural polysaccharide polymer, which has good biodegradability and can promote cell adhesion and growth (Adhikari et al., 2016), so the CCK-8 value further increased for Mg-Chi. After continuous surface modification, the surface hydrophilicity was enhanced and more chemical groups (such as–OH, –COOH, –NH_2_) were introduced, thus cell adhesion can also occur through non-receptor chemical binding, such as electrostatic, ionic–polar interactions, hydrogen binding to surface functional groups (Dekker et al., 1993; Liu et al., 2014b). After the introduction of PEG-Hep, the hydrophilicity became the best, which can provide excellent conditions for the initial growth of cells. Meanwhile, Mg-PEG-Hep has the best corrosion resistance and a non-polluting surface, which can inhibit protein adsorption and cell adhesion (Pan et al., 2016), and heparin can specifically bind to vascular endothelial growth factor to promote endothelial cells grow, so the CCK-8 value was the highest and the number of cells was also the highest. In summary, Mg-PEG-Hep had good cell compatibility, which was conducive to cell adhesion and growth.

## Conclusion

In this paper, PEG-Hep polymer was successfully synthesized and immobilized on the magnesium alloy, and its biological properties were evaluated by various methods. The introduction of PEG-Hep on the magnesium alloy can obtain the compact and dense layer, which can effectively enhance the corrosion resistance of the magnesium alloy. At the same time, due to the introduction of PEG-Hep, the blood compatibility of the magnesium alloy was significantly improved. In addition, the modified surface by PEG-Hep displayed good cytocompatibility to endothelial cells, the immobilization of PEG-Hep can significantly improve the adhesion and proliferation of vascular endothelial cells. In conclusion, the method of the present study can be used to modify the magnesium alloy to simultaneously improve the corrosion resistance biocompatibility, which can further enlarge the application of the magnesium alloy implants in the cardiovascular devices such as the stent.

## Data Availability

The original contributions presented in the study are included in the article/Supplementary Material, further inquiries can be directed to the corresponding author.
